# Analysis of codon usage bias of mitochondrial genome in *Bombyx mori* and its relation to evolution

**DOI:** 10.1186/s12862-014-0262-4

**Published:** 2014-12-17

**Authors:** Lei Wei, Jian He, Xian Jia, Qi Qi, Zhisheng Liang, Hao Zheng, Yao Ping, Shuyu Liu, Jingchen Sun

**Affiliations:** Subtropical Sericulture and Mulberry Resources Protection and Safety Engineering Research Center, Guangdong Provincial Key Laboratory of Agro-animal Genomics and Molecular Breeding, College of Animal Science, South China Agricultural University, Guangzhou, 510642 China; Guangzhou East Campus Lab Center, Sun Yat-sen University, Guangzhou, 510006 China

**Keywords:** *Bombyx mori*, Synonymous codon usage bias, Genomic DNA, Mitochondrial DNA, Evolution

## Abstract

**Background:**

Synonymous codon usage bias (SCUB) is an inevitable phenomenon in organismic taxa, generally referring to differences in the occurrence frequency of codons across different species or within the genome of the same species. SCUB happens in various degrees under pressure from nature selection, mutation bias and other factors in different ways. It also attaches great significance to gene expression and species evolution, however, a systematic investigation towards the codon usage in *Bombyx mori* (*B. mori*) has not been reported yet. Moreover, it is still indistinct about the reasons contributing to the bias or the relationship between the bias and the evolution of *B. mori*.

**Results:**

The comparison of the codon usage pattern between the genomic DNA (gDNA) and the mitochondrial DNA (mtDNA) from *B. mori* suggests that mtDNA has a higher level of codon bias. Furthermore, the correspondence analysis suggests that natural selection, such as gene length, gene function and translational selection, dominates the codon preference of mtDNA, while the composition constraints for mutation bias only plays a minor role. Additionally, the clustering results of the silkworm superfamily suggest a lack of explicitness in the relationship between the codon usage of mitogenome and species evolution.

**Conclusions:**

Among the complicated influence factors leading to codon bias, natural selection is found to play a major role in shaping the high bias in the mtDNA of *B. mori* from our current data. Although the cluster analysis reveals that codon bias correlates little with the species evolution, furthermore, a detailed analysis of codon usage of mitogenome provides better insight into the evolutionary relationships in Lepidoptera. However, more new methods and data are needed to investigate the relationship between the mtDNA bias and evolution.

## Background

Synonymous codon usage bias (SCUB) refers to the different frequency of synonymous codons in coding DNA. The triplets coding for the same amino acid are not equally used for protein expression, either among different organisms or the genes from a single species. The bias ensures that the most frequently used codons, the optimal codons, can pair with the anticodons of the most abundant tRNA genes [1]. And it also avoids the mis-incorporation of amino acids to reduce processing errors. It reveals a balance between natural selection (eg. translational selection, gene length, and gene function) and mutation bias (such as GC content and mutation position of base), as well as the influence of random genetic drift [2,3]. Therefore, understanding the codon usage bias can show the codon usage pattern of species, and provide evidence about the evolution of organisms. Many previous studies suggest that there are various factors related to SCUB, determined by mutational bias alone or by both mutation bias and natural selection [4]. For instance, the driving force of the bias in many mammals has been proved to be mutational bias [5], whereas some others believed that the natural selection determines the bias in eukaryotic organisms [6-8].

The domesticated silkworm, *Bombyx mori* (*B. mori*), which belongs to the Bombycidae family, is a well-studied model species of the Lepidopteran model system with a rich repertoire of genetic information about morphology, development, and behavior [9-11]. Previous studies investigating *B. mori* have primarily focused on the cloning, expression and characterization of some important genes [12-14], as well as applied research such as bioreactor [15] or antivirus [16-18]. In addition, there are also some studies on its evolution, which reveal that the domesticated silkworm derives from Chinese stock rather than Japanese or Korean stock [19-21]. With the completion of the whole genome sequence of *B. mori* [22-24], more molecular genetic resources can be used to illuminate the evolution history.

Originally, the classification of species was based on the observed morphology and behaviors. Later, a new taxonomic method called DNA barcoding was created that uses a short genetic marker in an organism’s DNA to identify it as belonging to a particular species [25,26]. However, it has been found that genes coded by genomic DNA (gDNA) seldom fit the standards of DNA barcode when compared to the genes from mitochondrial DNA (mtDNA). As the only genetic material outside gDNA, mtDNA is relatively small in size, extremely easy to amplify [27] and highly conservative, lacks extensive recombination, has maternal inheritance and shows a relatively high evolutionary rate and non-tissue specificity [28,29]. Thus, mtDNA is well used as a molecular marker in species identification, population genetics, systematic phylogeny and evolutionary studies [30-32].

The mtDNA of insects is a self-replicating, circular DNA molecule of about 14–20 kb in size, encoding a conserved set of 37 genes (13 protein genes, 22 tRNA genes, and 2 rRNA genes) and an A + T-rich segment known as control region [33,34]. Currently there are more than 260 species of insects whose mtDNA sequences have been determined (GenBank: www.ncbi.nlm.nih.gov/genbank). Hence, a combination of mtDNA and codon bias in *B. mori* might help us to understand the evolutionary relationships among the Lepidopteran species from another angle.

To test the above possibility, the primary aim of this study is to determine the factors contributing to the codon preference of mtDNA in *B. mori*. The results show that natural selection plays a major role in shaping the codon usage bias, while mutation bias only plays a minor role. Additionally, the evolutionary relationship of the species shown in our study is different from that of the traditional classification. A detailed and modified analysis based on mtDNA bias might offer a better understanding into the evolutionary relationships among the Lepidopteran insects.

## Results and discussion

### The codon usage pattern of gDNA and mtDNA in *B. mori*

The RSCU values and the number of each codon in the gDNA and mtDNA of *B. mori* are shown in Table 1. The codon usage frequency and the correspondent RSCU values were acquired from the Codon Usage Database (www.kazusa.or.jp/codon). It is found that codon preference happens both in the gDNA and the mtDNA. The GC content of the whole 1180 CDS’s (450, 043 codons) in the gDNA is 48.12%, while the values for the GC1s, GC2s and GC3s are 52.29%, 41.19%, and 50.89%, respectively. However, the GC content of 48 CDS’s (12, 937 codons) is 19.01% in mtDNA, and only 7.24% for the GC3s. Clearly, there are multiple differences within the GC contents between the gDNA and the mtDNA. And the frequency of codons ending with A/T is higher than that of G/C in the mtDNA. This may be due to the structure of the AT rich segments, and in turn leads to the higher codon bias.

**Table 1:** **The RSCU values and used codon numbers in the gDNA and mtDNA of** ***B. mori***

**AA**	**Codon**	**RSCU/number**		**AA**	**Codon**	**RSCU/number**	
		**gDNA**	**mtDNA**			**gDNA**	**mtDNA**
Phe	*TTT* ^*^	0.78/6894	*1.92/1439*	Tyr	*TAT* ^*^	0.78/6250	*1.88/641*
	**TTC**	**1.22/10823**	0.08/63		**TAC**	**1.22/9918**	0.12/42
Asn	*AAT* ^*^	0.90/9230	*1.76/790*	Cys	*TGT* ^*^	0.88/3929	*1.76/790*
	**AAC**	**1.10/11200**	0.24/108		**TGC**	**1.12/5094**	0.24/108
Lys	***AAA*** ^*^	**1.10/15283**	*1.84/414*	Arg	CGT	1.04/4399	0.72/58
	AAG	0.90/12759	0.16/37		CGC	1.14/4711	0.00/0
Leu	*TTA* ^*^	0.96/5885	*5.22/1640*		CGA	0.78/3153	1.26/97
	TTG	1.14/7124	0.36/114		CGG	0.60/2423	0.06/4
	CTT	0.84/5193	0.12/37		***AGA***	**1.50/6325**	*3.90/301*
	CTC	1.04/6570	0.00/0		AGG	0.96/4011	0.06/4
	CTA	0.66/3941	0.30/96	Ser	*TCT* ^*^	1.08/5718	*2.70/378*
	**CTG**	**1.32/8268**	0.00/3		TCC	1.022/5399	0.30/39
Pro	***CCT*** ^*^	**1.08/6283**	*1.80/165*		**TCA**	**1.08/5846**	2.04/292
	CCC	0.88/5256	0.44/42		TCG	0.90/4969	0.00/2
	CCA	1.04/6110	1.68/155		AGT	0.84/4632	0.90/128
	CCG	1.00/5860	0.08/8		AGC	1.02/5618	0.06/9
Thr	ACT	1.08/7037	1.40/163	Asp	*GAT* ^*^	0.94/11523	*1.90/176*
	ACC	1.00/6390	0.28/34		**GAC**	**1.06/12971**	0.10/9
	***ACA*** ^*^	**1.12/7146**	*2.24/265*	Glu	***GAA*** ^*^	**1.16/16217**	*1.80/216*
	ACG	0.84/5350	0.08/8		GAG	0.84/11781	0.20/24
Val	*GTT* ^*^	1.04/7558	*2.20/254*	Gly	**GGT**	**1.24/9761**	1.72/260
	GTC	1.04/7502	0.04/4		GGC	1.08/8443	0.16/22
	GTA	0.76/5588	1.72/195		*GGA* ^*^	1.24/9636	*1.80/275*
	**GTG**	**1.20/8735**	0.04/5		GGG	0.44/3465	0.32/51
Ala	***GCT*** ^*^	**1.40/11368**	*2.64/226*	Ile	*ATT*	1.02/8391	*1.77/1508*
	GCC	1.08/8860	0.20/16		**ATC**	**1.11/9321**	0.09/80
	GCA	0.80/6556	1.12/97		*ATA*	0.87/7173	1.14/956
	GCG	0.72/6066	0.04/4	Trp	TGG	1.00/5272	1.00/29
Met	ATG	1.00/10544	1.00/94	Gln	***CAA***	**1.04/8409**	*1.76/168*

Note: The preferentially used codons base on RSCU values for gDNA and mtDNA are in bold and italics, respectively. Asterisk (*) refers to the high frequency codons in mtDNA.

Normally, if the frequency value of a relative synonymous codon is over 60% or 1.5 times out to the average synonymous codon value of the group, it can be defined as the high frequency codon [35]. In the gDNA, only three high frequency codons (TTC, TAC and AGA) are found. At the same time, the other 19 preferentially used codons are also labeled (Table 1). However, 16 codons with high frequency are observed in the mtDNA. Among them, seven codons show a similar bias level, including AAA (Lys), CCT (Pro), ACA (Thr), GCT (Arg), AGA (Ala), GAA (Glu) and CAA (Gln). In the mtDNA, there are also some codons (eg. CTG, CCG, ACG, GTC, GTG, GCG, CGG, AGG, TCG, AGC and GAC) that are seldom used, and several codons which are never used, such as CTC, TAG and CGC (Table 1).

Our results reveal that the codon bias level of the gDNA is relatively lower than that of the mtDNA in *B. mori*. The high bias of the mtDNA might be derived from the evolution system of mitochondrion. As we know, the evolution patterns of mtDNA and gDNA are not identical. There are two main theories about the endosymbiotic origin of mitochondria at present. One of the theories suggests that the eukaryote engulfed the mitochondrion [36], while the other posits that the prokaryote host acquired the mitochondrion. As a relatively independent organelle inside the cell, early studies used to assume that mtDNA undergoes neutral evolution [37]. However, later findings have denied those findings, and it is now believed that mtDNA suffers from positive and negative selection [38-41]. An abundance of evidences supports the existence of co-evolution and co-adaption between the nuclear and mitochondrial genomes [42-49]. However, different substitution rates have been confirmed between them, where the mtDNA shows a higher rate of nucleotide substitution than that of the gDNA [50]. Thus, the higher rate of substitution might be related to the higher codon bias in the mtDNA.

### Correspondence analysis of codon bias

There are various reasons contributing to codon usage bias, including gene function, compositional constraints of genes, translational selection, the secondary structure of proteins, natural selection, and mutation bias [2,6,8]. Different substitutions suggest that mtDNA has experienced the non-uniform selective pressures [45]. Thus, the correspondence analysis of codons usage may help us to investigate the possible factors that cause the bias in the mtDNA of *B. mori.* Here, a set of indices were calculated, including G + C content, the content of bases in the third position (GC3s, G3s, T3s, C3s, and A3s etc.), and indices inflecting the codon bias level (eg. CBI, Fop, and ENc), as well as the gene function indices (eg. Gravy and Aromo).

Similar to the results of RSCU, the effective number of codons (ENc) in *B. mori* ranges from 23.14 to 44.00, with an average of 29.47, indicating a high-level codon bias in mtDNA. In addition, most of the tRNAs in mtDNA are known as inactive areas and have almost no ENc value, excluding the genes encoded tRNA-Thr, tRNA-Lys, tRNA-Ile. The absence of values might be a result of the extraordinarily short sequences which are unable to rightly encode enough amino acid, according to the definition of ENc and the calculation of CodonW [4].

**Figure 1:**
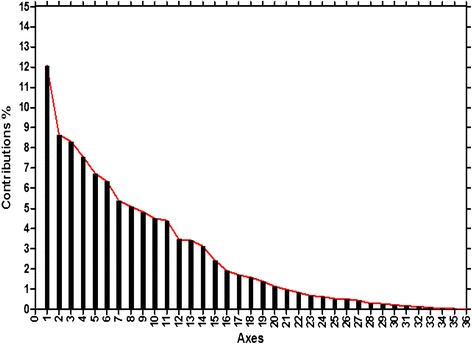
**Contributions of the axes.** Contributions of 36 axes were shown based on the COA.

**Figure 2:**
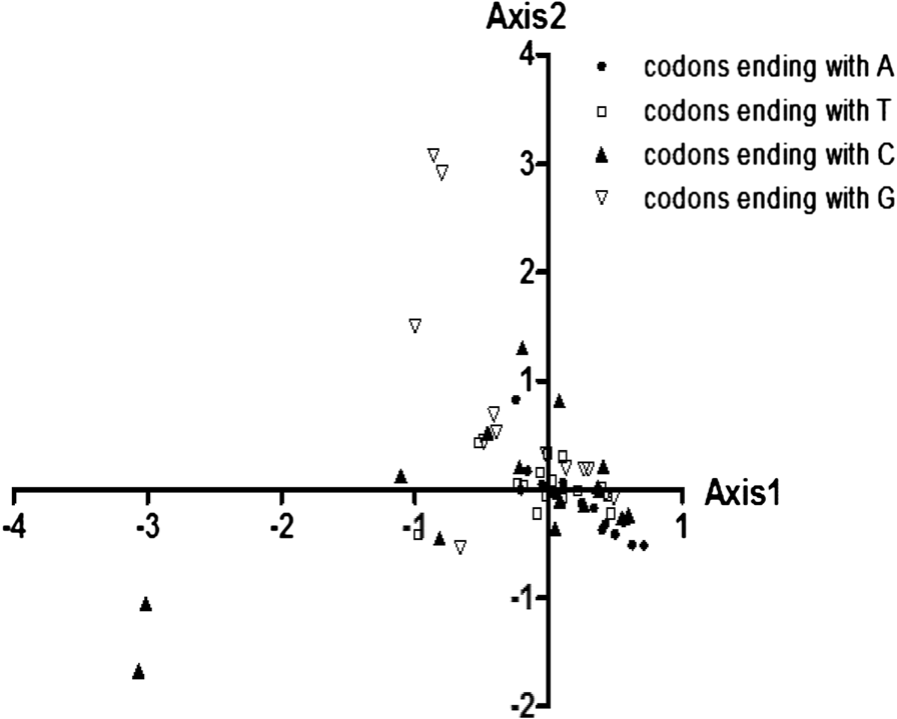
**Correspondence analysis of the synonymous codon usage towards the codons in mtDNA.** The analysis was based on the RSCU value of the 59 synonymous codons. The positions of each codon located in a super dimensional space were described in the first two-main-dimensional coordinates. Different base ended codons were marked in the figure, where the black point, white square, black triangle, white triangle refer to codons ending with A, T, C, G respectively.

**Figure 3:**
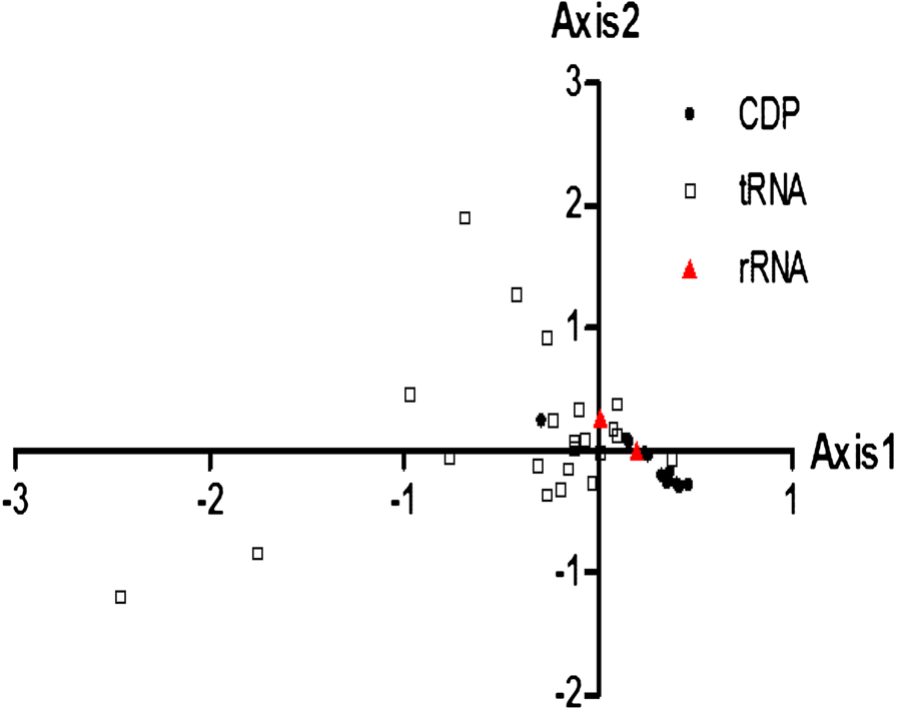
**Correspondence analysis of the synonymous codon usage towards the coding genes in mtDNA.** The analysis was based on the RSCU value of the 59 synonymous codons. The positions of each gene located in a super dimensional space were described in the first two-main-dimensional coordinates. Different genes were marked in the figure, where the black point, white square, red triangle refer to coding protein (CDP) gene, tRNA gene and rRNA gene respectively.

Based on the 59 synonymous codons, a set of 37 genes were represented by the points in a super dimensional axes and carried on to the correspondence analysis. Contributions of the axes were shown (Figure 1) and we arrived at a four main contributors which were Axis1, Axis2, Axis3 and Axis4. The first two-main-dimensional coordinates, Axis1 and Axis2 (Figure 2) can explain 12.07% and 8.64% of the total variation, while Axis3 and Axis4 explain 8.31% and 7.58%, respectively, which led to the first axis as the major contributor to the codon bias. The location of the genes marked in the two-dimensional coordinate after the correspondence analysis of 37 genes. The position of the AT ended codons are more close to Axis1 than the GC ending codons with a concentrate distribution (Figure 2), indicating that the base composition for mutation bias might correlate to the codon bias. While the genes with different GC content (CDP average 19.02%, tRNA average 19.61%, rRNA average 16.03%) showed a relatively regular distribution, and lower GC content gene located more close to Axis1 (Figure 3), implying that GC content for mutation pressure probably influenced the bias. In addition, a considerable amount of the genes are in a discrete distribution, indicating that there are many other factors that exist, for example, natural selection.

In order to intuitively display the indices related to the four main contributors, correlations of the important indices were calculated to determine the important factors that result in codon bias (Table 2).

**Table 2:** **Correlation coefficients of the factors that influence codon bias in mtDNA**

	**ENc**	**GC3s**	**GC**	**L_aa**	**Gravy**	**Axis1**	**Axis2**	**Axis3**
GC3s	**−0.389** ^*^							
GC	0.053	**0.351** ^*^						
L_aa	**0.649** ^**^	**−0.515** ^**^	0.063					
Gravy	**0.327** ^*^	**−0.505** ^**^	**−0.396** ^*^	**0.423** ^**^				
Axis1	**0.374** ^*^	**−0.581** ^**^	0.005	**0.455** ^**^	0.177			
Axis2	−0.032	0.143	0.121	**−**0.178	−0.015	0.120		
Axis3	**−0.365** ^*^	**0.393** ^*^	0.081	−0.285	**−0.335** ^*^	0.063	−0.045	
Axis4	−0.021	0.132	0.022	−0.086	0.052	**−**0.053	0.038	−0.070

Note: One asterisk (*) and two asterisks (**) indicate correlations at a level of 0.05 and 0.01, respectively.

Significant correlations are found in the indices. GC3s shows the significant correlation with Axis1 and ENc (r = −0.581, p < 0.01; r = −0.389, p < 0.05) suggesting that the base composition for mutation bias might have an impact on the codon bias. The first axis also shows the significant linear correlation with L_aa (r = 0.455, p < 0.01). Meanwhile, there is also a significant correlation between L_aa and ENc (r = 0.649, p < 0.01), revealing that gene length might affect the codon bias too. In addition, L_aa contains a negative correlation with GC3s (r = −0.545, p < 0.01), but strong positive linear correlation with ENc (r = 0.649, p < 0.01), which might give an assumption to the usage of synonymous codons suffered from the natural selection in the exact gene.

### Natural selection influences the codon bias of mtDNA

**Figure 4:**
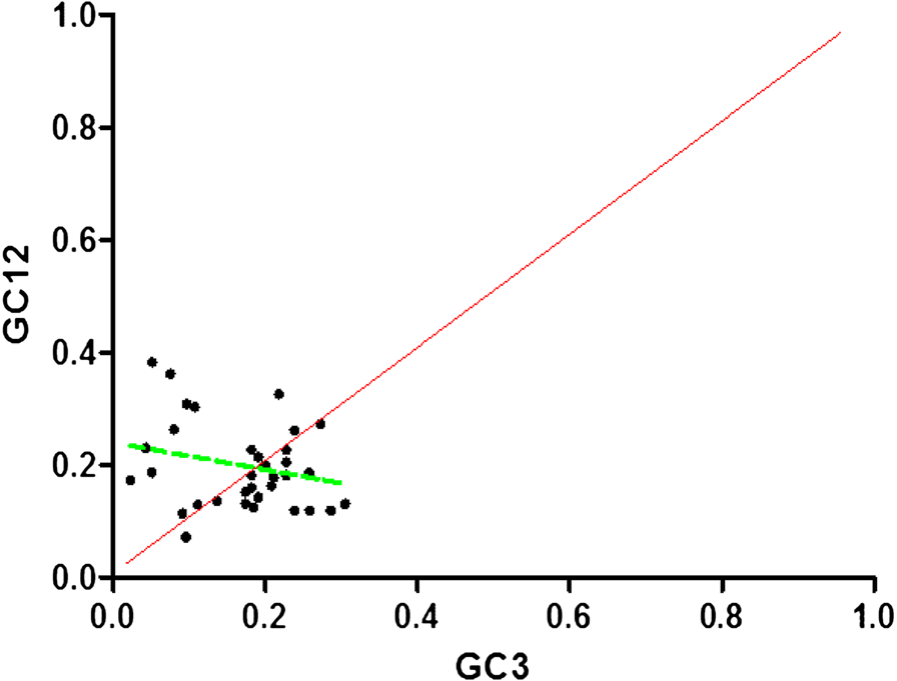
**GC12 against GC3 value of each gene.** GC12 stands for the average value of GC content in the first and second position of the codons (GC1 and GC2), while GC3 refers to the GC content in the third position. The diagonalin and the regression curve were colored in red and green, respectively. The regression curve can describe as y = −0.242x + 0.24, R^2^ = 0.0597, and GC12 showed no correlation with GC3.

A neutrality plot was drawn to estimate the extent of directional mutation pressure against selection in the codon usage bias of mtDNA. The neutrality plot (Figure 4) reveals the results of “equilibrium coefficient” of mutation and selection. GC12 is the average value of GC1 and GC2. The regression coefficient of GC12 to GC3 of mtDNA is 0.244 ± 0.074, indicating the relative neutrality is 24.40 % while the relative constraint is 75.60 % for GC3. In Figure 2, the points are not located to the diagonal distribution (red line) and the values of GC3 are in a narrow distribution, indicating GC12 and GC3 are definitely not the mutation bias model [25]. On the other hand, the regression curve (green line) tended to be sloped or parallel to the horizontal axis. The subsequent correlation analysis reveals little correlation between GC12 and GC3. Accordingly, mutation bias might just play a minor role in shaping the codon bias, whereas natural selection seems to probably dominate the codon bias.

### Mutation bias plays a minor role in the codon usage bias of mtDNA

**Figure 5:**
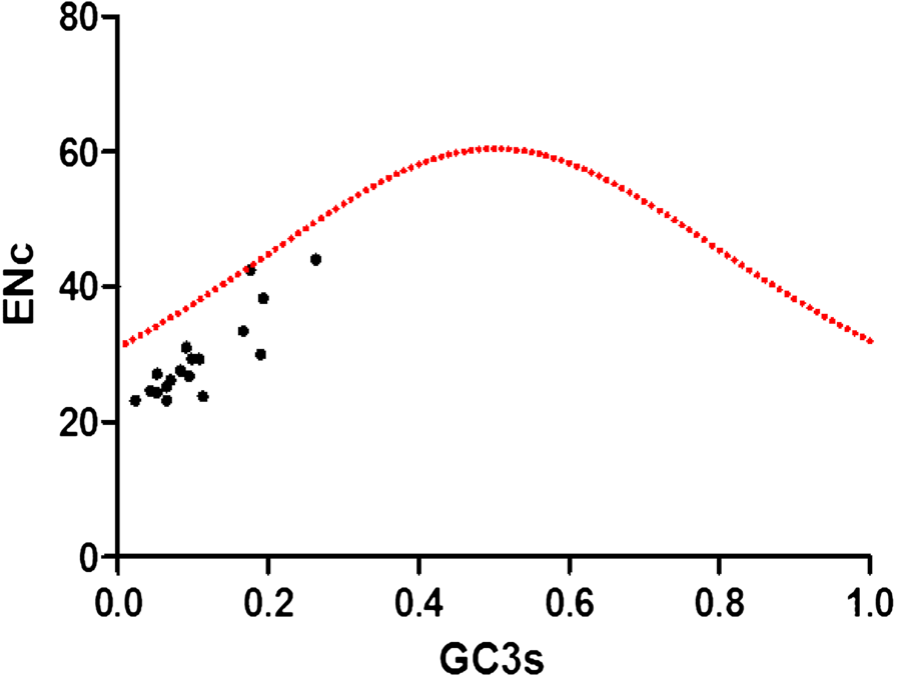
**ENc analysis of each gene plotted against GC3s.** The standard curve in red point in the figure showed the functional relation between ENc and GC3s is under mutation pressure without selection. Points on or close to the curve means bias caused by mutation pressure. Otherwise, they are affected by natural selection or other factors.

To further confirm whether mutation bias plays a relatively minor role in effecting the codon bias as deduced above, the ENc values were plotted against the GC3 values in ENc-plot [51] (Figure 5). The standard curve shows the functional relationship between ENc and GC3s is under mutation pressure rather than selection. In this case, if codon usage bias is completely based on GC3s, all the points will lie exactly on the standard curve (corresponding to the ENc values). For instance, the point of the gene 1-rRNA is in the plot just located on the curve, showing its codon bias is only or mainly affected by mutation pressure. However, most of the points do not lie close to the standard curve, which indicates that the GC3s for mutation bias is not the main factor in shaping the codon bias. For the points corresponding to different genes which show a discrete distribution, it implies other factors such as natural selection for gene length and gene expression level may also influence the codon bias to a certain extent.

It is generally acknowledged that codon preference reflects a balance between mutational bias and natural selection for translational optimization. In other words, natural selection and mutational bias are the two main factors leading to codon usage bias [52,53]. So far, combined with the neutrality plot, it shows that the SCUB of mtDNA is mainly dominated by natural selection, and mutational pressure only lightly affects the usage bias. Similarly, in many other species, such as *Arabidopsis thaliana*, *Drosophila melanogaster* or *Caenorhabditis elegans,* natural selection also plays a more important role in forming the codon bias of the whole genome [54,55].

### Gene length correlated to expression level causing the codon bias of mtDNA

**Figure 6:**
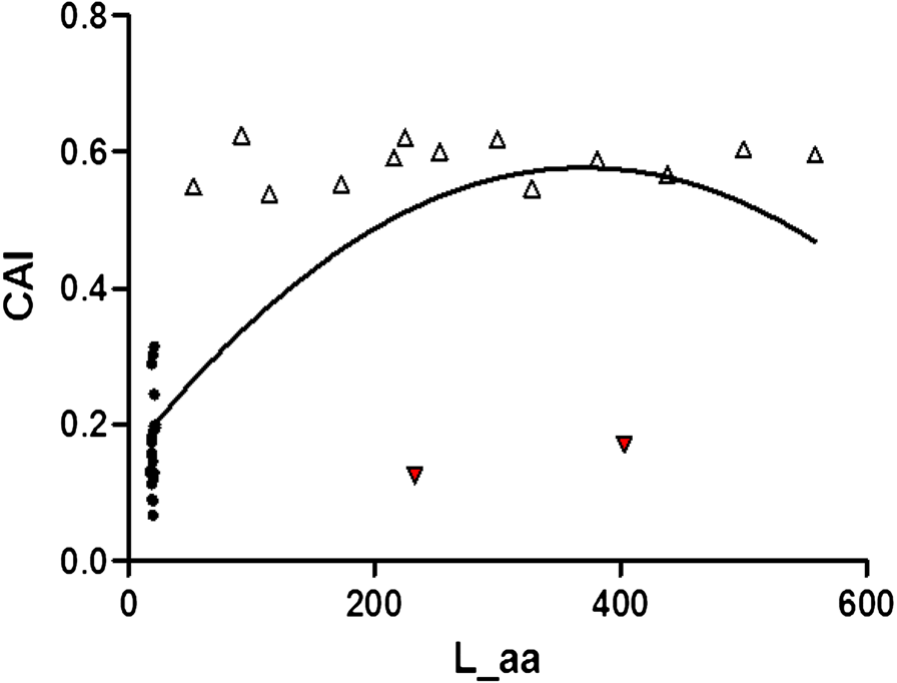
**CAI against gene length (L_aa value) of the coding genes.** The regression curve can be described as y = −3 × 10^−6^x^2^ + 0.0023x + 0.1514, R^2^ = 0.5694. The black point, white up triangle, and red triangle refer to tRNA gene, coding protein and rRNA gene respectively.

Ribosome genes of *B. mori* were used as a reference set to calculate the CAI value of the genes. Correlation analysis indicates that CAI is positive correlation against Axis1 (r = 0.504, p < 0.01) and strong negative correlation with ENc values (r = −0.851, p < 0.01). Thus, gene expression level is thought to be a factor for causing the bias. Furthermore, the correlation between CAI and gene length is significant (r = 0.683, p < 0.01) (Figure 6), indicating that gene length is related to expression level, although a few people believed that CAI and ENc were not correlated to the length of genes [56]. Additionally, the average CAI values of 13 protein-coded genes range from 0.5 to 0.7, a level close to 1.0, which might give an assumption that a relatively high expression level of the genes according to the index definition. The results are similar to previous studies [57]. However, it was necessary to investigate more data in order to identify that the 13 protein-coding genes in mitogenome of *B. mori* are highly expressed.

Both EST-based and new quantitative measures of gene expression (MPSS) suggest that codon preference is strongly associated with gene expression derived from the information on tRNA abundance [58]. Analysis about the bias might be of great importance to study gene expression, gene function, and even the evolution of the species since SCUB is considered to be an outcome of natural selection, mutation bias or genetic drift [59]. Thus, our analysis reveals that the high expression levels of the coded proteins making up the respiratory chain in mitochondrion meet well with the high energy demand to fit the life system. The process is recognized by the species containing abundant tRNA [60]. Large amount of tRNAs existing in the mitochondrion can also explain the high codon bias of mtDNA of *B. mori*. In addition, translational selection and translational efficiency also play a role in the natural selection towards codon bias. It is observed that long genes are highly expressed in *Yersinia pestis* [61], while the shorter genes have a better expression in *Drosophila melanogaster* [62,63]. This indicates the existence of translational selection and probably that the translational efficiency acts negatively in *Yersinia pestis,* but positively in *Drosophila melanogaster* in shaping the SCUB for the reason that high codon bias always appears in the highly expressed genes [6]. Our analysis suggests that gene length does play a role in shaping the bias and shows strong correlation with the gene expression level. Therefore we conclude that the translational efficiency is possibly a positive factor to cause the bias and the translational selection might also influence SCUB in mtDNA of *B. mori.*

Factors such as gene length, translational selection and translational efficiency can be due to the natural selection pressure that causes the SCUB in mtDNA of *B. mori.* The index Gravy correlated to the Axis3 might also be suggested as an aspect of the selection pressure in shaping the bias. However, indices which reflect the mutation pressure show little correlation with the primary axes, except GC3s. Combined with the neutrality plot and ENc- plot, the results suggest that it is natural selection rather than mutation pressure that dominates the SCUB in mtDNA of *B. mori.*

### The species classification based on the mtDNA SCUB

**Figure 7:**
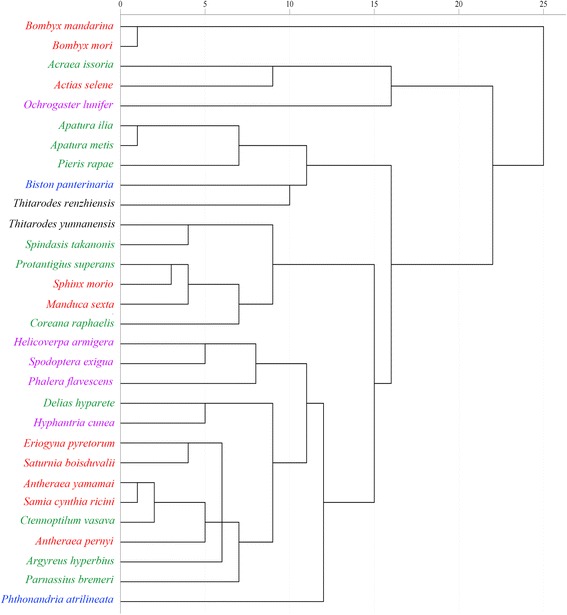
**Cluster analysis of the 30 species in Lepidoptera based on the codon usage value of mtDNA.** Where five superfamilies are in different colors: Bombycoidea (red), Papilionoidea (green), Noctuoidea (purple), Geometroidea (blue), Hepialoidea (black).

30 Neolepidoptera species were used for the cluster analysis, included in five superfamilies (Bombycoidea, Geometroidea, Hepialoidea, Noctuoidea, and Papilionoidea) and 13 families, using the RSCU values from correspondence analysis of the 13 CDSs in each mitogenome. The cluster analysis to *Bombyx* and other species with a relatively near genetic distance in Lepidoptera reveals that all the species are divided into two big clusters at the evolution distance of about 23 (Figure 7). *B. mori* and *Bombyx mandarina* stay the closest and are isolated almost from the root*.* While the other species in the Bombycoidea family known as have close relationship with the *Bombyx* are divided into another branch. Thus another branch is divided into several clades in turn. However, species in the same color are in a discrete distribution, although several small numbers of species gather together. The results appear largely different from the previous studies [64].

However, almost all of the species in different families are in a similar mean value of ENc, GC content and GC3s (Table 3). Hence, we can hardly classify the species according to the codon usage indicies, except the clade of *B. mori* and *Bombyx mandarina* which has extremely lower ENc value from the other species. Thus, the codon usage of mitogenome might trace a different evolutionary path from the way of species evolution. It could be also assumed that codon bias might determine the mitochondrial genome in a specific way and transcend the boundary of phylogenetic relationships of species [65].

**Table 3:** **List of taxa in the phylogenetic and correspondence analysis with their GenBank accession numbers**

**Species**	**GenBank No.**	**ENc ± SD**	**GC3s% ± SD**	**GC% ± SD**
*Bombyx mandarina*	NC_003395	27.70 ± 2.04	7.08 ± 0.02	19.13 ± 0.05
*Bombyx mori*	NC_002355	27.73 ± 2.72	7.23 ± 0.03	19.35 ± 0.05
*Actias selene*	NC_002355	32.87 ± 2.51	10.32 ± 0.02	21.57 ± 0.05
*Antheraea pernyi*	NC_018133	31.26 ± 4.05	7.20 ± 0.03	20.22 ± 0.06
*Antheraea yamamai*	NC_012739	30.46 ± 2.83	6.17 ± 0.02	19.51 ± 0.06
*Eriogyna pyretorum*	NC_012727	30.08 ± 2.50	5.62 ± 0.02	18.98 ± 0.06
*Samia cynthia ricini*	NC_017869	31.30 ± 1.87	7.92 ± 0.02	20.23 ± 0.06
*Saturnia boisduvalii*	NC_010613	31.20 ± 2.58	6.64 ± 0.02	19.25 ± 0.06
*Manduca sexta*	NC_010266	28.92 ± 2.68	5.12 ± 0.02	18.43 ± 0.05
*Sphinx morio*	NC_020780	30.18 ± 2.48	6.75 ± 0.03	19.25 ± 0.04
*Biston panterinaria*	NC_020004	30.79 ± 3.33	7.78 ± 0.02	19.60 ± 0.05
*Phthonandria atrilineata*	NC_010522	30.19 ± 2.87	7.72 ± 0.03	19.43 ± 0.06
*Thitarodes yunnanensis*	NC_018095	28.89 ± 1.69	4.46 ± 0.01	17.82 ± 0.05
*Thitarodes renzhiensis*	NC_018094	31.81 ± 211	8.57 ± 0.03	19.58 ± 0.05
*Hyphantria cunea*	NC_014058	30.24 ± 2.32	8.18 ± 0.01	20.10 ± 0.05
*Helicoverpa armigera*	NC_014668	30.10 ± 1.98	6.65 ± 0.02	19.36 ± 0.05
*Spodoptera exigua*	NC_019622	29.66 ± 2.49	5.83 ± 0.02	19.30 ± 0.05
*Ochrogaster lunifer*	NC_011128	33.80 ± 3.22	14.63 ± 0.03	23.02 ± 0.06
*Phalera flavescens*	NC_016067	31.47 ± 4.08	7.24 ± 0.04	19.93 ± 0.06
*Ctenoptilum vasava*	NC_016704	31.43 ± 3.51	7.33 ± 0.02	19.49 ± 0.06
*Coreana raphaelis*	NC_007976	29.06 ± 2.17	3.44 ± 0.02	17.27 ± 0.04
*Protantigius superans*	NC_016016	29.41 ± 2.77	5.11 ± 0.02	18.28 ± 0.05
*Spindasis takanonis*	NC_016018	29.19 ± 2.03	5.44 ± 0.02	17.61 ± 0.05
*Apatura ilia*	NC_016062	30.99 ± 2.90	8.60 ± 0.03	19.67 ± 0.05
*Apatura metis*	NC_015537	31.45 ± 2.69	8.48 ± 0.02	19.64 ± 0.05
*Argyreus hyperbius*	NC_015988	30.79 ± 3.33	7.78 ± 0.02	19.60 ± 0.05
*Parnassius bremeri*	NC_014053	30.65 ± 3.97	5.66 ± 0.02	18.61 ± 0.05
*Delias hyparete*	NC_020428	31.68 ± 3.03	7.72 ± 0.03	20.02 ± 0.06
*Pieris rapae*	NC_015895	31.90 ± 2.14	8.94 ± 0.02	20.20 ± 0.06
*Acraea issoria*	NC_013604	32.65 ± 1.20	9.78 ± 0.03	20.52 ± 0.05

Note: All of the ENc, GC3s%, GC% are mean values.

## Conclusions

Many factors can result in the synonymous codon usage bias of organisms. For the mtDNA of *B. mori*, natural selection is found to dominate the high SCUB. And we believe that mutation bias only plays a relatively minor role. Moreover, our study provides new insight into the exploration of setting up new methods for species taxonomy, though a trial still needs to be conducted in the future.

## Methods

### Codon usage indices

For codon bias, both mutational pressure and natural selection are two core factors. The first step is to assess the codon bias level in analysis, and then determine which one is the driving force. At present, many statistical methods have been proposed and all the common indices are utilized in our study.

#### Measurement indices of codon bias level

##### Effective number of codons (ENc)

It is often used to measure the codon bias in one specific gene [51], which is an assessment of non-uniformity of usage within synonymous groups of codons. ENc values can vary from 20 (extreme bias where only one codon is used per amino acid) to 61 (without bias where codons are randomly used).

##### Relative synonymous codon usage (RSCU)

This index for a particular codon is given by the observed frequency divided by the expected frequency. If all synonymous codons coding the same amino acid are used equally, RSCU values are close to 1.0, indicating a lack of bias.

##### Codon bias index (CBI) and frequency of optimal codons (Fop)

They are another two common indicators for assessing codon bias [66].

#### Indices related with mutation bias

##### GC3s

It presents the frequency of use of G + C in the synonymously variable third positions of the sense codon (i.e., excluding Met, Trp and termination codons).

#### Indices related with natural selection

##### Codon adaptation index (CAI)

The CAI index uses a reference set of highly expressed genes from a species to assess the relative merits of each codon, and a score for a gene is calculated from using the frequency of all codons in that gene. The index assesses the level to which selection has been effectively molding the pattern of codon usage. The CAI values can range from 0 to 1, and the larger ones reveal a higher gene expression level as well as codon bias. In that respect, CAI is useful for predicting the expression level of a particular gene [67].

##### Length of synonymous codons (L_sym) and length of amino acids (L_aa)

The two indices represent the number of synonymous codons and the number of translatable codons, respectively.

##### General average hydropathicity (GRAVY) and Amoro

The GRAVY index is used for the hypothetical translated gene product, calculated as the arithmetic mean of the sum of the hydropathic indices of each amino acid [68]. Its score range from −2 to 2: positive value shows hydrophobic protein, while negative one indicates hydrophilic protein. Amoro refers to aromaticity, which represents the frequency of aromatic amino acids (Phe, Tyr, Trp) in the hypothetical translated gene product. Both the hydropathicity and aromaticity protein scores are indices of amino acid usage. In *E. coli* genes, the strongest trend in the variation in the amino acid composition is correlated with protein hydropathicity, the second trend is correlated with gene expression, and the third is correlated with aromaticity [69]. The variation in amino acid composition can influence the analysis results of codon usage.

### Multivariate statistical analysis

Several methods for the multivariate statistical analysis were used here:

#### Principal component analysis (PCA)

This method is usually used to analyze genes by site in a 59-dimensional space based on the RSCU values [70]. PCA can transform some of large correlated variables (eg. RSCU values) into major smaller ones. PCA was used here to find the factors determining the major trends in codon usage bias, such as mutation bias or natural selection.

#### Correspondence analysis (COA)

It is utilized to compare and analyze two or more categories of variable data in one contingency table. Variable categories (rows and the columns) are presented in a low dimension space diagram, in which relatively simple data is displayed to replace the original data, eliminating excess noise and complex data structure, and providing visual results [71-73].

#### Linear regression analysis (LRA) and factor analysis (FA)

The two methods were also used to show the correlations of the indices and the codon usage pattern of the mtDNA in *B. mori*’s.

#### Cluster analysis (CA)

In the analysis, data is classified into different categories according to their similarities. The clustering analysis to *B. mori* was made among 8 representative organisms using the method of “squared Euclidean distance”. And the final results came from the clustering analysis of the most debated classification between Bombycidae and Saturniidae based on the whole mtDNA sequence and the codon frequency in mitochondrial, respectively.

#### ENc-plot mapping analysis

The analysis was employed in order to find out the determing factor in influencing the codon usage bias, in which the ENc values were plotted against the GC3s values in Nc-plot [51]. The standard curve shows the functional relation between ENc and GC3s was under mutation pressure rather than selection. In this case, if the codon usage is optimal, the analyzed points will lie on or just below the expected curve (composed of ENc values). Otherwise, genes with serious codon bias and significant correlation with gene expression will be low in ENc values and show points far lower than the standard curve.

#### Neutrality plot mapping analysis

The frequent mutations of the synonymous codons usually happen in the third position, whereas some mutations occurring in the first or the second positions are non-synonymous. The frequency of these non-synonymous mutations are relatively small, caused by the gene function or gene activity. This means that if there is no outside pressure, mutations should occur in random rather than in a certain direction under the condition of pressure. Thus, there is no difference between three codon positions, and base content composition is also similar. However, bases preferences of the three positions are different in the presence of certain selection pressures [74]. When drawing, GC12 works as the ordinate while GC3 works as the abscissa, and each dot represents an independent gene. If all the points lie along the diagonal distribution, it indicates that no significant difference exists on the three codon positions, and there is no or only a weak external selection pressure. On the other hand, if the regression curve tends to be sloped or parallel to the horizontal axis, it means that the variation correlation between GC12 and GC3 is very low. Therefore, the regression curve works well to measure the neutral degree when selecting effect dominates the evolution [74,75].

### Software used

All indices of codon usage bias above were calculated in the data set using the program CodonW 1.4.4 (http://codonw.sourceforge.net/) [4]. Clustering analysis and correlation analysis were carried out using the statistical software SPSS 19.0. Graphs were generated in GraphPad Prism 5.0 (http://www.graphpad.com/scientific-software/prism/).

### Availability of supporting data

The codon usage of the 48 CDSs (12937 codons) in mtDNA and the 1180 CDSs (450043 codons) in the gDNA in Table 1 were downloaded from the Codon Usage Database (http://www.kazusa.or.jp/codon/). The codon usage of *B. mori* ribosome used in calculating the CAI value was also downloaded from the Codon Usage Database. The sequence set of the 37 genes in the mtDNA of *B. mori* (NC_002355) used in correspondence analysis were obtained from GenBank (http://www.ncbi.nlm.nih.gov/genbank). And all of the mtDNA CDSs of the 30 species used in clustering analysis were achieved from GenBank.

## Abbreviations

SCUB: Synonymous codon usage bias
gDNA: Genomic DNA
mtDNA: Mitochondrial DNA
CDS: Coding sequence
CDP: Coding protein
ENc: Effective number of codons
RSCU: Relative synonymous codon usage
CAI: Codon adaptation index
CBI: Codon bias index
Fop: Frequency of optimal codons
GRAVY: General average hydropathicity
Amoro: Amoromaticity
PCA: Principal component analysis
COA: Correspondence analysis
LRA: Linear regression analysis
FA: Factor analysis
Lei Wei: Jian He and Xian Jia contributed equally to this work
